# Advancements in Biologic Therapies for Pediatric Asthma: Emerging Therapies and Future Directions

**DOI:** 10.7759/cureus.83629

**Published:** 2025-05-07

**Authors:** Pablo Xavier Anda Suárez, Uziel Márquez Romero, Nayely García Méndez, María José Rengel Chalco, María Alejandra Vivas Monzón, Ciro Gonzalo Zavala Gama

**Affiliations:** 1 Primary and Outpatient Care, Universidad de Las Américas, Quito, ECU; 2 Intensive Care - Emergency, Hospital de Alta Especialidad de Veracruz, Veracruz, MEX; 3 Medical Sciences, Universidad de La Frontera, Temuco, CHL; 4 General Practice - Surgery, Sanatorio Santa Bárbara, Universidad de Buenos Aires (UBA), Buenos Aires, ARG; 5 Pediatrics, Universidad Francisco Marroquín, Guatemala City, GTM; 6 Pediatrics, Red de Salud Arequipa Caylloma, Arequipa, PER

**Keywords:** advancement, biologic therapy, medicine-pediatrics, pediatric asthma, severe asthma

## Abstract

This review aims to analyze biologic treatments for pediatric asthma, along with their effects on T2-high inflammation, together with outcomes of omalizumab, mepolizumab, and dupilumab. This review examined recent biomarker advancements, together with endotype patterns, while analyzing early treatment opportunities that might transform asthma's natural course. In conclusion, the advancement in biologic therapies is offering significant progress for personalized severe asthma treatment among pediatrics, especially for the T2-high endotype. Biologics such as omalizumab, mepolizumab, dupilumab, benralizumab, and tezepelumab have been reported to reduce severe asthma exacerbations, with reassuring short-term safety profiles among children and adolescents (pediatrics).

However, the treatment efficacy of these biologics is limited for T2-low and non-T2 asthma endotypes, emphasizing the need for new biologic therapies that target these endotypes. Literature review also highlights the emerging treatment regimens for non-T2 endotypes, such as tezepelumab, ecleralimab, and astegolimab (IL-33), which influence both T2 and non-T2 pathways. The integration of precision medicine and multi-omics data specific to patients is not only providing promising results, but also helping to refine patient selection and patient-specific treatment. Furthermore, the identification of novel predictive biomarkers through advanced omics methods is essential for a more personalized approach. Ultimately, the continued advancement and strategic implementation of biologic therapies hold the potential to revolutionize the management of severe pediatric asthma, leading to reduced exacerbations and corticosteroid use, improved quality of life, and more precise treatment strategies tailored to individual patient profiles. Moreover, future research should continue to address the challenges related to long-term safety, cost-effectiveness, and equitable access, which are vital to realizing the true potential of biologic therapies in treating pediatric asthma.

## Introduction and background

Pediatric asthma is a common chronic respiratory disease that significantly affects children globally. It is characterized by airway inflammation and hyperreactivity, leading to symptoms such as wheezing, coughing, and shortness of breath. Asthma is the most common chronic disease in children [1]. In 2019, the global prevalence of asthma in individuals aged 5-69 years was estimated to be between 5% and 17.8% in 220 population-based studies [2]. Global Burden of Disease (GBD) collaborators predicted that approximately 260 million individuals had poorly managed current asthma (diagnosed asthma with wheeze within 12 months) in 2019, causing disability and early deaths in numerous low- and middle-income countries [3]. Current asthma prevalence varies over two decades. After rising to 8.5% in 2003 and 9.6% in 2009, it fell to 7.0% in 2019. Male prevalence (9.9%) was consistently greater than female prevalence (7.5%) [4]. Older children aged 10-17 (10.4%) had a greater asthma prevalence than younger children aged 0-4 (5.3%) and 5-9 (9.5%). A racial difference was also observed, as Black children (14.3%) had more asthma than White (7.6%) and Asian (5.4%) children [4].

Many children with asthma can be controlled with low to medium doses of inhaled corticosteroids (ICS), while 5% to 10% have severe asthma [5]. Childhood severe asthma harms patients and society; it can cause frequent symptoms, asthma exacerbations, lung function impairment, and lower quality of life (QoL) [6]. Black and Hispanic youngsters in the U.S. have more severe asthma exacerbations. The hospitalizations and deaths caused by asthma exacerbations are significant. Systemic corticosteroids, used to treat severe exacerbations, might cause serious side effects after one dose. Also, severe asthma can impair lung function and small airway hyperresponsiveness [7].

Traditional asthma management follows a stepwise approach, primarily using ICS and bronchodilators [8]. While effective for many, a subset of children with severe asthma remains uncontrolled despite high doses of ICS and other controller medications. For these children, there is a strong need to adapt asthma treatment to the individual patient, considering underlying inflammatory profiles, and moving towards a personalized approach [9].

For many severely asthmatic children, biologic treatments are a crucial add-on [10]. Biologics have been available for adults and adolescents for nearly 20 years, but research on their efficacy and safety in younger children has lagged, but has lately expanded. Monoclonal antibodies target immune system components, including asthma inflammatory cascade cytokines [11].

Rationale

The rationale for exploring biologics in severe pediatric asthma stems from the understanding that current biologics target T2 inflammatory phenotypes in many severe asthmatic children. By targeting specific mediators of this inflammation, such as IgE, IL-5, IL-4, and IL-13, biologics aim to reduce severe asthma exacerbations, improve symptom control, and potentially reduce the need for systemic corticosteroids, thus mitigating their associated risks [12]. The heterogeneity of severe pediatric asthma, with different underlying inflammatory patterns (endotypes), further supports the need for targeted biologic therapies to achieve better outcomes and move towards precision medicine in pediatrics.

Objective and scope of review

This review aims to analyze biologic treatments for pediatric asthma, along with their effects on T2-high inflammation, together with outcomes of omalizumab, mepolizumab, and dupilumab. This review examines recent biomarker advancements, together with endotyping strategies, while analyzing early treatment opportunities that might transform asthma's natural course. The review analyzed both short-term and long-term safety aspects and health outcomes, including treatment accessibility and costs. The research examines upcoming therapeutic targets that extend past T2 pathways by studying IL-6 and IL-17. The review scope focused on analyzing current evidence and identifying a way forward for future research that can optimize biologic therapeutic treatments for asthmatic children. The literature review was conducted, focusing on the last five years of all study designs retrieved from peer-reviewed journals related to the topic of discussion. 

## Review

Current landscape of biologic therapies

The current landscape of biologic therapies in pediatric asthma is characterized by a shift towards personalized medicine, recognizing the heterogeneity of the disease, and targeting specific inflammatory pathways in pediatrics.

Overview of Asthma Endotypes

Pediatric asthma is a complex inflammatory disease. While asthma presents with common clinical features, the underlying inflammatory patterns (endotypes) can differ among pediatric patients. The asthma inflammatory pathways are T2 (allergic/eosinophilic), T2-low (pauci-granulocytic and less allergic/eosinophilic), and T1/17 (neutrophilic). Asthma's varied immunological responses (T2, T17, T1, innate) emphasize illness heterogeneity, yet only biologic anti-T2 therapies have been effective [12]. Many asthma endotypes are T2-high or T2-low.

T2-inflammatory endotype: Childhood asthma is characterized by T helper type 2 (Th2) inflammation. Th2 inflammation is generally characterized by aeroallergen sensitization, enhanced peripheral blood eosinophils, and high fractional exhaled nitric oxide (FeNO) levels. Childhood asthma is characterized by high total serum IgE, blood eosinophilia, and aeroallergen polysensitization. Current biologics target Th2 phenotypes, which are found in most severe asthmatic children [13]. Eosinophilic airway inflammation and high IL-4, IL-5, and IL-13 levels define T2-high asthma, which can be allergic or non-allergic, depending on atopy. T2-high airway inflammation is commonly indicated by blood eosinophil levels >150 or 300 cells/μL, and a percentage of exhaled nitric oxide (FeNO, >25 ppb) [14]. Many biologic therapies have been identified and well-documented to treat T2-high endotypes, which will be discussed ahead.

T2-low inflammatory endotype: T2-low asthma has no T2-high biomarkers and can be separated into T17-high asthma (neutrophilic airway inflammation, and high IL-17 and IL-22 levels) and T2-low/T17-low asthma [14]. However, it is important to note that there is limited or no efficacy of biologic therapies to treat T2-low asthma in pediatrics. There is a need for more treatment options for T2-low phenotypes.

Non-T2 endotypes: Non-T2 endotypes are characterized by inflammation driven by neutrophils, Th1, and Th17 cells. Neutrophilic (IL-17 and IL-6 promote neutrophilic recruitment), pauci-granulocytic (low levels of neutrophils and eosinophils), and mixed granulocytic asthmas are non-T2 subtypes. The diagnosis of non-T2 asthma is challenging due to the absence of T2 biomarkers and steroid resistance. Physicians often rely solely on exclusion criteria to diagnose it. Therefore, understanding the molecular mechanisms of IL-17 and IL-6 is essential for improving diagnosis and treatment [15]. Thus, evidence for biologic therapies is limited to nonexistent for non-T2 types.

Summary of Currently Approved Biologics

Monoclonal antibodies targeting specific inflammatory mediators, such as T2 endotype pathways, have been approved for children and adolescents with severe asthma.

Omalizumab: It is a humanized anti-IgE monoclonal antibody, which is suitable for children six years and older with moderate to severe persistent allergic asthma. Omalizumab treatment is needed for those patients with increased serum IgE levels, a positive skin test, or in vitro reactivity to a perennial aeroallergen, and poor symptom control despite using ICS. In teenagers above 12, FEV1 may need to be decreased. Omalizumab prevents free serum IgE from binding to mast cells and basophil IgE receptors, blocking pro-inflammatory mediator release. It downregulates IgE receptors and cell-bound IgE. Omalizumab, the first asthma biologic, has the most safety and effectiveness data [16].

Mepolizumab: This is an anti-IL-5 monoclonal antibody that is approved for children six years and older with severe eosinophilic asthma. Mepolizumab neutralizes IL-5, a cytokine that promotes eosinophil proliferation, differentiation, mobilization, activation, recruitment, and survival. This selectively inhibits eosinophilic inflammation and lowers sputum and blood eosinophils [17,18].

Dupilumab: This monoclonal antibody blocks IL-4 and IL-13 signaling by targeting the IL-4 receptor α-subunit. It is approved for teenagers ≥12 years with severe T2 inflammation and children ≥6 years whose asthma is uncontrolled despite high-dose ICS and other maintenance medications. Increased IL-4, IL-5, IL-13, and IgE production characterize asthma T2 inflammation. Dupilumab lowers Th2 inflammation in eosinophilic asthma and allergies [19].

Benralizumab: The drug is an anti-IL-5 receptor α monoclonal antibody. It is approved for adolescents older than 12 years with severe eosinophilic asthma. Eosinophils and basophils die from antibody-dependent cell-mediated cytotoxicity when benralizumab binds to their IL-5 receptors. It leads to a rapid and near-complete elimination of eosinophils in blood and sputum [18].

Tezepelumab: This monoclonal antibody targets anti-thymic stromal lymphopoietin (TSLP). It has been approved for severe asthma in adults and children ≥12 years, without phenotypic or biomarker restrictions. Tezepelumab, the first biologic without phenotype and biomarker restrictions for severe asthma, affects airway inflammation via TSLP [20].

Reported Clinical Outcomes

All these approved biologics have demonstrated the ability to reduce the rates of severe asthma exacerbations.

Omalizumab: It has shown a reduction in exacerbations and hospital admissions, as well as a decrease in overall corticosteroid use in children with moderate to severe allergic asthma. In a systematic review by Hanania et al., omalizumab reduced exacerbations, improved lung function, asthma control, QoL, health care resource utilization, and corticosteroid (oral/inhaled) use in adult patients with moderate-to-severe allergic asthma for up to nine years. Pediatric patients treated for up to 7.5 years with omalizumab had similar results. This research validates the long-term therapeutic advantages of omalizumab in pediatric and adult allergic asthma patients [21].

Mepolizumab: Treatment in patients with severe eosinophilic asthma has been shown to significantly reduce asthma exacerbations. It also demonstrates steroid-sparing effects and may improve asthma control and QoL, although improvements in lung function might be less consistent. Jackson et al. carried out a multicenter randomized controlled trial. The 290 participants were randomly assigned to mepolizumab (n = 146) or placebo (n = 144) with intention-to-treat analysis. The 248 participants stayed until the study was finished. An asthma exacerbation averaged 0.96 with mepolizumab and 1.30 with placebo in the 52-week study (rate ratio 0.73; 0.56-0.96; p = 0.027). Treatment-emergent adverse events occurred in 42 (29%) of 146 mepolizumab participants and 16 (11%) of 144 placebo participants. No mepolizumab patients died during or after therapy [22].

Dupilumab: It has shown efficacy in reducing severe exacerbations in adolescents and children with T2 inflammation. It has also demonstrated a steroid-sparing effect in glucocorticoid-dependent severe asthma. Dupilumab might offer more consistent improvements in lung function compared to other biologics. In a randomized controlled trial, Corren et al. found that dupilumab 200/300 mg every two weeks reduced severe asthma exacerbation rates (-36.9%/-45.5%; both p < 0.01) and improved FEV1 at week 12 in LIBERTY ASTHMA QUEST patients with or without allergic asthma (total serum IgE ≥30 IU/mL and ≥1 perennial aeroallergen-specific IgE ≥0.35 kU/L). Patients with high T2 inflammatory biomarkers were more effective, leading to improved asthma control. Dupilumab quickly and consistently reduced T2 inflammation. Similar results were shown in non-allergic asthmatics (n = 819) [23].

Benralizumab: It has been shown to reduce exacerbation rates in severe eosinophilic asthma in adolescents. It also decreased the oral corticosteroid (OCS) daily dose, ensuring certainty of evidence. Wedner et al. included 28 children aged 6-11 and two Japanese children aged 12-14 in their study. Near-complete blood eosinophil depletion was observed across dose/weight groups. In exploratory efficacy studies, mean FEV1, patient and clinician global impression of change, and exacerbation rates improved numerically. No participant died from 78.6% of adverse outcomes. Benralizumab is safe and effective for long-term use in children with severe eosinophilic asthma, including teenagers and adults [24].

Tezepelumab: It demonstrated a significant decrease in annual asthma exacerbations in adolescents and adults with severe, uncontrolled asthma, including those with low eosinophil counts. Pavord et al. conducted NAVIGATOR, a multicenter, double-blinded, randomized controlled trial to examine the effect of tezepelumab on asthma exacerbations across all seasons in NAVIGATOR patients. Tezepelumab reduced exacerbations in severe, uncontrolled asthmatics with seasonal and perennial allergies vs. placebo across all seasons. These findings suggest the efficacy of tezepelumab in a wide range of severe, uncontrolled asthmatics [25].

While all approved biologics generally reduce exacerbation rates, their effect on pulmonary function may be less consistent and may vary. Some biologics, like dupilumab, might offer more pronounced improvements in lung function [26].

Novel insights and emerging domains

Biomarkers and Endotyping

Identifying related biomarkers and endotypes has been of growing interest among researchers and specialists, because there is a shift toward precision medicine, necessitating a better understanding of inflammatory patterns. This shift aims to move away from a 'one size fits all' approach to more personalized treatments [27].

Role of Multi-omics Approaches

Multi-omics approaches, which integrate data from genomics, epigenomics, transcriptomics, proteomics, metabolomics, microbiomics, and exposomics (environmental exposures), are providing new insights into the complexity of childhood asthma and potential therapeutic targets. Advanced molecular classification may improve asthma phenotyping and subgrouping. Systems medicine can incorporate multiple omics methods to improve molecular phenotyping [28,29]. Large consortia, such as Unbiased Biomarkers for the Prediction of Respiratory Disease Outcome (U-BIOPRED), Severe Asthma Research Program (SARP), and Systems Pharmacology Approach to Uncontrolled Pediatric Asthma (SysPharmPediA), are focused on unraveling the complex pathophysiology of uncontrolled asthma using multi-omics studies. Abdel-Aziz et al. aim to use multi-omics systems medicine to study pathophysiological pathways in moderate-to-severe uncontrolled and managed asthma patients on maintenance medication. This multicenter observational case-control study comprised 6- to 17-year-old Dutch, German, Spanish, and Slovenian moderate-to-severe asthmatics. Subjects were grouped by asthma exacerbations and control. Clinical, demographic, and patient/family histories were obtained. Multiple samples were examined for epigenomics, transcriptomics, microbiome, proteome, and metabolomics. In this sample of 145 children, 91 had uncontrolled asthma (median age = 12 years, 43% girls), and 54 had controlled asthma (median age = 11.7 years, 37% girls). Groups had similar age, sex, and BMI z-score distributions. Details and noninvasive biosample methodologies for omics studies will reveal pathophysiological pathways in moderate-to-severe uncontrolled pediatric asthma. Unique biomarkers for noninvasive diagnosis and treatment may be identified [30].

Role of Noninvasive Diagnostics

Noninvasive diagnostics are particularly valuable in children, as collecting sputum specimens can be difficult, and venipunctures can cause distress.

Fractional exhaled nitric oxide (FeNO): FeNO is a marker of airway inflammation, primarily reflecting IL-13 activity and T2 inflammation. In adults, higher baseline FeNO is related to better clinical outcomes from dupilumab, but its significance in predicting response to other biologics, such as mepolizumab and benralizumab, is less clear. Omalizumab responders have decreased baseline FeNO. Responses to omalizumab in pediatric patients showed only a non-significant decrease in FeNO. Age-dependent FeNO reference values in children complicate interpretation [31].

Nasal swabs: Upper airway samples are taken using nasal swabs. Upper airway transcriptome profiles from nasal samples predicted disease activity and biologic therapy response better than blood eosinophils and FeNO, in a recent mepolizumab trial in urban children and adolescents. Molecular phenotyping, employing such approaches, is crucial for individualized treatment [22]. Furthermore, a longitudinal study in children aged 5-11 years found an association between upper airway microbiota obtained from nasal samples and severe exacerbation rates.

Metabolomics measurements in exhaled breath (breath omics): Breath omics, which analyze volatile organic compounds (VOCs), may be a simple, noninvasive way to identify asthma phenotypes in children and guide treatment. VOCs can distinguish neutrophilic and eosinophilic asthma and predict exacerbations. Research on biological responses from exhaled breath is ongoing [32].

Refining Patient Selection Based on Genetic and Proteomic Profiles

More accurate inflammatory asthma endotype identification is feasible thanks to molecular technology. Some asthma phenotypes respond differently to certain treatments.

Genetic predisposition: It plays a significant role in the development of asthma, with most genes causing predisposition by regulating epithelial barrier function and innate and adaptive immune responses. Genome-wide association studies (GWAS) have identified several genes associated with asthma. Pharmacogenetics aims to understand how genetic variations influence drug response, potentially allowing for more tailored prescribing of asthma medications, including biologics [33]. This area needs more research, especially in children affected by asthma.

Proteomic profiles: Proteomic profiles, the large-scale study of proteins, can provide insights into the active biological pathways in asthma. By analyzing protein expression patterns in different asthma endotypes, it may be possible to identify biomarkers that predict response to specific biologics [34]. For example, serum periostin levels have been suggested as a biomarker for T2-high asthma in adults and may be associated with airway remodeling. However, in children, periostin is influenced by growth and age, limiting its reliability as a biomarker [35].

The integration of multi-omics data, including genetic and proteomic information, with clinical and conventional biomarker data holds the promise of refining patient selection for biologic therapies and moving towards true precision medicine in pediatric asthma [28-30,33,35]. Novel biomarkers and more extensive biomarker profiles are essential for predicting treatment response and selecting the best biologic for each patient. However, translating these advanced omics technologies into routine clinical practice is difficult due to cost, computational resources, and the need for multidisciplinary collaboration. We need additional studies on pediatric asthma, as molecular pathways, clinical outcome impacts, biomarkers, and biological responses are age-dependent. Most studies have focused on adults.

Early intervention and disease modification with biologics in pediatric asthma

Evidence and Potential for Biologics to Alter Asthma’s Natural History

Current literature suggests that there is emerging interest in whether biologics can modify the natural history of childhood asthma. In adults, discontinuing biologics usually returns asthma activity to pretreatment levels, but in children, lung growth, development, and puberty changes may allow biologics to be discontinued after several years of therapy. However, specific research is needed to identify suitable candidates [11].

The Preventing Asthma in High-Risk Kids' Study (PARK, NCT 02570984) is the first to test biologics' disease-modifying effects by seeing if omalizumab treatment in preschoolers at high risk for asthma prevents childhood asthma. This highlights the potential interest in using biologics for early intervention to alter the course of the disease. The rationale is that early intervention, particularly with therapies blocking IL-4 and IL-13, might be attractive in childhood asthma, which is often driven by allergen sensitization, eosinophilia, and T2 immunity. It is also considered important to investigate the impact of biologics on airway remodeling, as this can impact lung function, even in very young children [36].

The recent studies intend to explore the potential role of biologics in altering the disease course, and the findings will be considered hopeful. Moreover, similar future studies are also suggested to explore this aspect further with more longitudinal trials.

Seasonal or Short-Term Biologic Use in Preventing Exacerbations

Seasonal omalizumab prevents exacerbations. A study of 478 urban children, aged 6 to 17, with at least one exacerbation in the preceding year revealed that starting omalizumab medication before autumn school admission and using guideline-based care decreased fall asthma exacerbations by 52%. This suggests a potential role for short-term, targeted use of certain biologics, like omalizumab, to prevent predictable seasonal increases in asthma exacerbations [11,34,37].

Overall, while long-term data on disease modification are still needed - especially in the pediatric population - the existing evidence points toward the potential for early intervention and targeted, short-term use of biologics like omalizumab to influence the natural history and prevent seasonal exacerbations in childhood asthma. Further research is crucial to determine which patients are most likely to benefit from these strategies and the optimal duration of treatment.

Long-term safety and immunomodulatory effects of biologics in pediatric asthma

Gaps in Long-Term Data for Younger Children

Current literature highlights the lack of long-term biologic safety evidence in children, especially younger ones. Clinical studies lasting one year or less provide most of the safety data. Approved biologics, including omalizumab, mepolizumab, and dupilumab, have promising short-term safety profiles, but long-term trials are required to assess their effectiveness and safety in pediatric populations.

Long-term evidence is lacking; hence, Saglani et al. recommend follow-up to understand the effects of long-term biologic usage in children on the developing immune system [38]. van Dijk et al. note that pediatric efficacy and safety evidence is sparse, especially for recently approved biologics like dupilumab and tezepelumab [26]. Agache et al. explained, adding that only omalizumab and mepolizumab have long-term safety data in children, but other biologics need to be further explored to determine long-term efficacy [39]. Ferrante et al. recommend more studies on long-term dupilumab treatment to assess long-term adverse effects [40]. While omalizumab is generally well-tolerated, Votto et al. recommend long-term monitoring studies to confirm its safety [41]. Okobi et al. further note that there is no child-focused, evidence-based asthma diagnosis and management guideline, which often leads to the extrapolation of adult data [42].

Considerations Regarding Immune Development and Vaccination Response

The developing immune system in children is a crucial consideration when using immunomodulatory therapies like biologics. Saglani et al. note that the immune system develops postnatally and may have different immunological processes in early life than in adulthood, making extrapolating adult data to children difficult. Age matters when utilizing biologics that target critical immune pathways because allergen exposure during this 'window of opportunity' can affect the immune response and pathology [38]. Furthermore, Saglani et al. raise a critical point about the potential long-term consequences of biologic use on a developing immune system. Since cells like eosinophils, targeted by some biologics, have roles in homeostasis and immunity, the long-term effects of their depletion are unclear. This information is particularly relevant when considering biologics as treatments for childhood asthma [38].

Okobi et al. remark that tests used in adult asthma patients may not be as effective or appropriate in young patients, and the ideal cutoffs may differ. This evidence suggests that the immunological landscape and responses to interventions can vary with age [42]. While some sources mention the potential for biologics to improve antiviral immunity, there is no specific information in the provided sources addressing the impact of biologics on vaccination responses in children with asthma. This remains an area where further research is necessary to ensure that children receiving these therapies maintain adequate protection against vaccine-preventable diseases.

In conclusion, there are significant gaps in long-term safety data for biologics in younger children with asthma. The ongoing development of the immune system in children necessitates careful consideration of the potential long-term effects of immunomodulatory therapies. The provided sources do not offer specific information regarding the impact of biologics on vaccination responses in this population, highlighting another area requiring further investigation.

Health equity and economic considerations in pediatric asthma

Disparities in Access and Outcomes Across Socioeconomic and Racial/Ethnic Groups

Several articles highlight disparities in asthma, including severe asthma, among different socioeconomic and racial/ethnic groups.

Black and Hispanic youngsters in the U.S. have more severe asthma exacerbations. In the 2018 U.S. pediatric asthma prevalence report, Puerto Ricans (17%) and non-Hispanic Blacks (14.3%) had higher rates than the national 7.5% for children under 18. Low-income households (10.2%) also had a higher prevalence. Urban residence and neighborhood poverty are linked to higher asthma morbidity among children on Medicaid [5,34].

A European PERMEABLE survey indicated large variations in biologics treatment per site for children. This evidence shows that location and doctor experience may affect access. The report also found that national rules and child therapy requirements caused considerable access variations throughout Europe. Some countries had few eligible children due to national health insurance system reimbursement rules. The survey emphasizes the need for healthcare system harmonization to improve biologic access for European children with severe asthma [43].

Cost-Effectiveness and Resource Utilization in Pediatric Care

The economics of pediatric asthma biologics are also examined. Even though severe asthma affects less than 5% of children with asthma, it accounts for 50% of their healthcare costs. These patients need extensive diagnostics and healthcare resources. The high cost of biologic medications is important [40].

A systematic review found that all biologics (benralizumab, dupilumab, mepolizumab, omalizumab, and reslizumab) for severe eosinophilic asthma in adults have incremental cost-effectiveness ratios (ICERs) per quality-adjusted life years (QALYs) above the willingness to pay. This economic research had low bias but high imprecision and indirectness, resulting in intermediate certainty. Hospitalizations, emergency room (ER) visits, and primary care visits decrease with biologic use, potentially saving money. Administration expenses should decrease with most biologics' autoinjector approval. Some research indicated very high ICER values for benralizumab, dupilumab, mepolizumab, and reslizumab in terms of cost-effectiveness. Day-to-day health-related QoL, asthma-related mortality, biologic acquisition price, and temporal horizon determine cost-effectiveness. Biologics are expensive; hence, they will likely be used only in severe uncontrolled asthma patients to reduce exacerbation rates or OCS use [39]. Long-term safety and cost-effectiveness evaluations, including real-world studies, registries, and big data analysis, are needed. Home biologic administration reduces hospital visits, healthcare expenses, and professional stress while improving patient and caregiver convenience.

A study by Makhecha et al. showed that self-administration of biologics at home for asthmatic children (6-18 years), with remote supervision and monitoring, is safe and well-received [44]. Integrating pharmacoeconomics into clinical decision-making is suggested to ensure optimal resource utilization. Collaborative studies and the collection of real-world data are essential for benchmarking and addressing unmet needs in practice, including the cost-effective selection of different biologics. In summary, significant disparities exist in pediatric asthma regarding prevalence, exacerbation rates, and access to advanced therapies such as biologics across socioeconomic and racial/ethnic groups, as well as geographic locations. Economic evaluations indicate that biologics are costly, with ICER values often exceeding the willingness-to-pay thresholds, although they can lead to savings by reducing hospitalizations and emergency care. Further research on cost-effectiveness in the pediatric population, and strategies to improve equitable access, are needed.

Future therapeutic targets beyond T2 pathways

Current literature indicates an ongoing exploration of non-T2 inflammatory pathways in asthma to identify potential biologic targets beyond the current focus on T2 inflammation. While T2 inflammation, driven by cytokines like IL-4, IL-5, and IL-13, is common in childhood asthma, non-T2 asthma, characterized by neutrophilic or pauci-granulocytic patterns, also exists. This non-T2 inflammation is promoted by cytokines such as IL-8, IL-17, and IL-22, as well as epithelial cell-derived cytokines.

Literature highlights the following points regarding future therapeutic targets beyond T2 pathways: tezepelumab, an anti-TSLP monoclonal antibody, is already approved for severe asthma in patients above 12 years, and, importantly, it is the first asthma biologic without restriction to specific biomarkers or inflammatory mechanisms of severe asthma. TSLP is an epithelial-derived alarmin cytokine that upregulates both T2 and non-T2 (i.e., IL-17) cytokine production. Clinical trials for tezepelumab showed a significant decrease in annual asthma exacerbations, irrespective of baseline T2 inflammatory status. There is a recognized need for more treatment options, specifically for patients with a T2-low asthma phenotype.

Inhaled anti-TSLP monoclonal antibody ecaleralimab may reduce systemic side effects and decrease doses. Another biologic in development may treat T2-high and T2-low asthma: astegolimab, a human monoclonal antibody that suppresses IL-33 signaling by targeting its receptor, ST2. Airway epithelial cells generate IL-33, an alarmin that activates Th1, Th2, and other lymphoid cells after tissue injury. A study found that astegolimab reduced asthma exacerbations, regardless of phenotype [26].

While IL-17 is known to be involved in non-T2 asthma, the sources do not provide specific information about anti-IL-17 biologics currently in the incidence of asthma. The ongoing research aims to better understand the mechanisms driving both T2-high and T2-low endotypes, to identify novel therapeutic targets and predictive biomarkers. Advances in omics technologies may help in discovering non-invasive markers reflecting distinct underlying inflammatory patterns. In summary, the field is moving beyond solely targeting T2 pathways in severe asthma. TSLP and IL-33 are key players in both T2 and non-T2 inflammation, and biologics targeting these alarmins, such as tezepelumab, astegolimab, and ecaleralimab, represent promising future therapeutic options for a broader range of severe asthma patients, including those with non-T2 phenotypes. Further research is crucial to fully elucidate the role of other non-T2 cytokines, like IL-17, in asthma pathogenesis and to develop targeted therapies for these pathways.

## Conclusions

In conclusion, the advancement in biologic therapies is offering significant progress for pediatric severe asthma treatment, especially for the T2-high endotype. In children and adolescents, biologics such as omalizumab, mepolizumab, dupilumab, benralizumab, and tezepelumab prevent severe asthma exacerbations with excellent short-term safety. These biologics have limited efficacy for T2-low and non-T2 asthma endotypes, highlighting the need for novel biologic treatments. The literature review also discusses developing non-T2 endotype treatments, such as tezepelumab, ecaleralimab, and astegolimab (IL-33), which affect both T2 and non-T2 pathways. Precision medicine and patient-specific multi-omics data are yielding encouraging findings and improving patient selection and treatment. A more tailored strategy requires improved omics approaches to identify novel prognostic biomarkers. The development and strategic use of biologic therapies could revolutionize severe pediatric asthma management, reducing exacerbations and corticosteroid use, improving QoL, and providing more personalized treatment strategies. Standardized management methods for pediatrics with severe asthma are also needed. Moreover, future research should continue to address the challenges related to long-term safety, cost-effectiveness, and equitable access, which are vital to realizing the true potential of biologic therapies in treating pediatric asthma.

## References

[1] Lizzo JM, Goldin J, Cortes S (2025). Pediatric asthma. StatPearls [Internet].

[2] Song P, Adeloye D, Salim H, Dos Santos JP, Campbell H, Sheikh A, Rudan I (2022). Global, regional, and national prevalence of asthma in 2019: a systematic analysis and modelling study. J Glob Health.

[3] Vos T, Lim SS, Abbafati C (2020). Global burden of 369 diseases and injuries in 204 countries and territories, 1990-2019: a systematic analysis for the Global Burden of Disease Study 2019. Lancet.

[4] Ojo RO, Okobi OE, Ezeamii PC (2023). Epidemiology of current asthma in children under 18: a two-decade overview using National Center for Health Statistics (NCHS) data. Cureus.

[5] Bacharier LB, Maspero JF, Katelaris CH (2021). Dupilumab in children with uncontrolled moderate-to-severe asthma. N Engl J Med.

[6] Crossingham I, Turner S, Ramakrishnan S (2021). Combination fixed-dose beta agonist and steroid inhaler as required for adults or children with mild asthma. Cochrane Database Syst Rev.

[7] Lugogo N, Judson E, Haight E, Trudo F, Chipps BE, Trevor J, Ambrose CS (2022). Severe asthma exacerbation rates are increased among female, Black, Hispanic, and younger adult patients: results from the US CHRONICLE study. J Asthma.

[8] Cazzola M, Matera MG, Rogliani P, Calzetta L, Ora J (2021). Step-up and step-down approaches in the treatment of asthma. Expert Rev Respir Med.

[9] Reddel HK, Bacharier LB, Bateman ED (2022). Global initiative for asthma strategy 2021: executive summary and rationale for key changes. J Allergy Clin Immunol Pract.

[10] Perikleous EP, Steiropoulos P, Nena E, Paraskakis E (2022). Biologic therapies in pediatric asthma. J Pers Med.

[11] Bacharier LB, Jackson DJ (2023). Biologics in the treatment of asthma in children and adolescents. J Allergy Clin Immunol.

[12] Lindsley AW, Lugogo N, Reeh KA, Spahn J, Parnes JR (2025). Asthma Biologics across the T2 spectrum of inflammation in severe asthma: biomarkers and mechanism of action. J Asthma Allergy.

[13] Papadopoulos NG, Bacharier LB, Jackson DJ (2024). Type 2 inflammation and asthma in children: a narrative review. J Allergy Clin Immunol Pract.

[14] Brusselle GG, Koppelman GH (2022). Biologic therapies for severe asthma. N Engl J Med.

[15] Liu T, Woodruff PG, Zhou X (2024). Advances in non-type 2 severe asthma: from molecular insights to novel treatment strategies. Eur Respir J.

[16] Berghea EC, Balgradean M, Pavelescu C, Cirstoveanu CG, Toma CL, Ionescu MD, Bumbacea RS (2021). Clinical experience with anti-IgE monoclonal antibody (omalizumab) in pediatric severe allergic asthma - a Romanian perspective. Children (Basel).

[17] Antosz K, Batko J, Błażejewska M, Gawor A, Sleziak J, Gomułka K (2024). Insight into IL-5 as a potential target for the treatment of allergic diseases. Biomedicines.

[18] Jackson DJ, Wechsler ME, Brusselle G, Buhl R (2025). Targeting the IL‐5 pathway in eosinophilic asthma: a comparison of anti‐IL‐5 versus anti‐IL‐5 receptor agents. Allergy.

[19] Le Floc'h A, Allinne J, Nagashima K (2020). Dual blockade of IL-4 and IL-13 with dupilumab, an IL-4Rα antibody, is required to broadly inhibit type 2 inflammation. Allergy.

[20] Shinkai M, Yabuta T (2023). Tezepelumab: an anti-thymic stromal lymphopoietin monoclonal antibody for the treatment of asthma. Immunotherapy.

[21] Hanania NA, Niven R, Chanez P, Antoine D, Pfister P, Garcia Conde L, Jaumont X (2022). Long-term effectiveness and safety of omalizumab in pediatric and adult patients with moderate-to-severe inadequately controlled allergic asthma. World Allergy Organ J.

[22] Jackson DJ, Bacharier LB, Gergen PJ (2022). Mepolizumab for urban children with exacerbation-prone eosinophilic asthma in the USA (MUPPITS-2): a randomised, double-blind, placebo-controlled, parallel-group trial. Lancet.

[23] Corren J, Castro M, O'Riordan T (2020). Dupilumab efficacy in patients with uncontrolled, moderate-to-severe allergic asthma. J Allergy Clin Immunol Pract.

[24] Wedner HJ, Fujisawa T, Guilbert TW (2024). Benralizumab in children with severe eosinophilic asthma: pharmacokinetics and long-term safety (TATE study). Pediatr Allergy Immunol.

[25] Pavord ID, Hoyte FC, Lindsley AW (2023). Tezepelumab reduces exacerbations across all seasons in patients with severe, uncontrolled asthma (NAVIGATOR). Ann Allergy Asthma Immunol.

[26] van Dijk YE, Rutjes NW, Golebski K, Şahin H, Hashimoto S, Maitland-van der Zee AH, Vijverberg SJ (2023). Developments in the management of severe asthma in children and adolescents: focus on dupilumab and tezepelumab. Paediatr Drugs.

[27] Vijverberg SJ, Brinkman P, Rutjes NW, Maitland-van der Zee AH (2020). Precision medicine in severe pediatric asthma: opportunities and challenges. Curr Opin Pulm Med.

[28] Gautam Y, Johansson E, Mersha TB (2022). Multi-omics profiling approach to asthma: an evolving paradigm. J Pers Med.

[29] Radzikowska U, Baerenfaller K, Cornejo-Garcia JA (2022). Omics technologies in allergy and asthma research: an EAACI position paper. Allergy.

[30] Abdel-Aziz MI, Neerincx AH, Vijverberg SJ (2021). A system pharmacology multi-omics approach toward uncontrolled pediatric asthma. J Pers Med.

[31] Murugesan N, Saxena D, Dileep A, Adrish M, Hanania NA (2023). Update on the role of FeNO in asthma management. Diagnostics (Basel).

[32] Malik M, Demetrowitsch T, Schwarz K, Kunze T (2024). New perspectives on 'Breathomics': metabolomic profiling of non-volatile organic compounds in exhaled breath using DI-FT-ICR-MS. Commun Biol.

[33] Ridolo E, Pucciarini F, Nizi MC, Makri E, Kihlgren P, Panella L, Incorvaia C (2020). Mabs for treating asthma: omalizumab, mepolizumab, reslizumab, benralizumab, dupilumab. Hum Vaccin Immunother.

[34] Alizadeh Bahmani AH, Abdel-Aziz MI, Maitland-van der Zee AH, Vijverberg SJ (2022). Recent advances in the treatment of childhood asthma: a clinical pharmacology perspective. Expert Rev Clin Pharmacol.

[35] Kumar K, Singh M, Mathew JL, Vaidya PC, Verma Attri S (2023). Serum periostin level in children with asthma. Indian J Pediatr.

[36] Phipatanakul W, Mauger DT, Guilbert TW (2021). Preventing asthma in high risk kids (PARK) with omalizumab: design, rationale, methods, lessons learned and adaptation. Contemp Clin Trials.

[37] Fenu G, La Tessa A, Calogero C, Lombardi E (2022). Severe pediatric asthma therapy: omalizumab - a systematic review and meta-analysis of efficacy and safety profile. Front Pediatr.

[38] Saglani S, Yates L, Lloyd CM (2023). Immunoregulation of asthma by type 2 cytokine therapies: treatments for all ages?. Eur J Immunol.

[39] Agache I, Song Y, Alonso-Coello P (2021). Efficacy and safety of treatment with biologicals for severe chronic rhinosinusitis with nasal polyps: a systematic review for the EAACI guidelines. Allergy.

[40] Ferrante G, Tenero L, Piazza M, Piacentini G (2022). Severe pediatric asthma therapy: dupilumab. Front Pediatr.

[41] Votto M, De Filippo M, Licari A, Marseglia A, De Amici M, Marseglia GL (2021). Biological therapies in children and adolescents with severe uncontrolled asthma: a practical review. Biologics.

[42] Okobi OE, Okoronkwo CA, Duru H, Iyayi IR, Adeakin-Dada TO, Doherty NO (2024). A review of the latest guidelines for diagnosing and managing asthma in children in the United States and Canada. Cureus.

[43] Santos-Valente E, Buntrock-Döpke H, Abou Taam R (2021). Biologicals in childhood severe asthma: the European PERMEABLE survey on the status quo. ERJ Open Res.

[44] Makhecha S, Jamalzadeh A, Irving S (2021). Paediatric severe asthma biologics service: from hospital to home. Arch Dis Child.

